# The Tryptophan-Kynurenine pathway in people living with HIV: a systematic review

**DOI:** 10.1007/s15010-025-02557-1

**Published:** 2025-05-31

**Authors:** Tshiamo Will Sebigi, Levanco K. Asia, Grant G. January, Esmé Jansen van Vuren, Monray Edward Williams

**Affiliations:** 1 Biomedical and Molecular Metabolism Research (BioMMet), North-West University, Potchefstroom, South Africa; 2 School of Biomedical Sciences, University of Plymouth, Plymouth, Devon UK; 3 Hypertension in Africa Research Team (HART), North-West University, Potchefstroom, South Africa; 4 South African Medical Research Council Unit for Hypertension and Cardiovascular Disease, North-West University, Potchefstroom, South Africa

**Keywords:** Metabolomics, Metabolite dysregulation, Antiretroviral therapy, Immunometabolism, Biomarkers

## Abstract

**Purpose:**

HIV-1 disrupts the metabolic profile of people living with HIV (PLWH), including the Tryptophan–Kynurenine (Trp–Kyn) pathway, linked to disease outcomes and comorbidities. Despite numerous studies, consensus on key dysregulated metabolites in antiretroviral therapy (ART)-treated PLWH is lacking. This systematic review compiles data to identify and highlight the most noteworthy Trp–Kyn metabolites.

**Methods:**

PubMed, Scopus, and Web of Science databases were searched using a search protocol specifically designed for this study. Studies that investigated the levels of metabolites in the Trp–Kyn pathway in the peripheral blood of PLWH on ART, as well as in healthy control groups were included.

**Results:**

Thirteen metabolomic studies that investigated this pathway met our inclusion criteria. The findings revealed that Trp, Kyn, and the Kyn/Trp ratio (indicative of indoleamine 2,3-dioxygenase IDO activity) were the most investigated metabolites in this metabolic pathway. Evidence consistently demonstrated that Trp levels were lower in PLWH, while predicted IDO activity was consistently higher. Despite the widespread investigation of Kyn, there was no clear consensus on its levels in PLWH, with some studies reporting higher levels and others finding no significant differences compared to HIV-negative controls.

**Conclusion:**

In the modern ART era, Trp metabolism and IDO activity may play key regulatory roles in HIV-1 pathogenesis, as evidenced by the consistent patterns observed across various studies. These metabolites and related pathways warrant further investigation as potential targets for improved diagnostics, prognostics, and therapeutics in the context of HIV-1.

**Supplementary Information:**

The online version contains supplementary material available at 10.1007/s15010-025-02557-1.

## Introduction

Human immunodeficiency virus (HIV) remains one of the most significant global health challenges, with an estimated 39 million of people living with HIV (PLWH) globally, with approximately 37.5 million being adults and 1.5 million children aged 0–14 years [1]. Despite extensive awareness campaigns and prevention efforts, nearly 1.8 million new infections were reported in 2021 [1]. Due to the high genetic variability of the virus, HIV can be categorised into two main types, HIV-1 and HIV-2, both responsible for the development of acquired immunodeficiency syndrome (AIDS) [2]. The primary clinical distinction between the two types of HIV infection is the rate of immunodeficiency progression, as HIV-2 has a slower rate of progression compared to HIV-1 [2]. Furthermore, HIV-2 is geographically predominant in West Africa and Europe, while HIV-1 is prevalent throughout the world [2]. Due to significant health implications (higher CD4 decline, higher viral loads, lower median survival rates) related to HIV-1 [3], this strain is more commonly investigated [4]. HIV-1 is classified into four groups: M (major), N (non-M/non-O), O (outlier), and P. Group M is the most prevalent globally, responsible for the vast majority of HIV-1 infections worldwide [5]. In contrast, Group O accounts for only a few thousands of infections, primarily in West and Central Africa, while Group N has been identified in only a small number of PLWH, mostly in Cameroon [6]. Both Group M and Group N originated independently but directly from SIVcpz, a simian immunodeficiency virus found in the chimpanzee species, Pan troglodytes troglodytes, native to West-Central Africa [6]. HIV-1 group M can be further divided into subtypes: A, B, C, D, F, G, H, J, and K based on variants in the viral genome [7].

With the availability of treatments like combination antiretroviral therapy (cART), HIV can be effectively managed. When administered and utilized correctly, cART can ultimately prevent the progression to AIDS [8]. Even in the modern cART era, several pathophysiological pathways remain dysregulated, often altering clinical outcomes in PLWH. These include chronic immune activation and inflammation [9, 10], gut dysbiosis [11], HIV-associated neurocognitive disorders (HAND) [12], and metabolic complications [13]. Evidence suggest that PLWH experience persistent metabolic changes despite the effective use of ART, with various pathways being influenced [14].

Of particular interest for this review is the Tryptophan–Kynurenine (Trp–Kyn) pathway, as dysregulation in the metabolites of this pathway has been implicated in adverse clinical outcomes (disease progression, immunological suppression, T-cell disruption and dysbiosis of the gut microbiota) in PLWH [15–18]. Tryptophan (Trp) is one of the essential amino acids in the body that the body cannot synthesize, and as a result, Trp must be obtained through diet [19, 20]. In the human body, Trp is mainly metabolized by four major pathways namely tryptamine, serotonin, indole-3-pyruvate and kynurenine (Kyn). However, the body converts majority of Trp to Kyn [21]. This metabolic shift is catalyzed by three enzymes: Indoleamine 2,3-dioxygenase 1 (IDO-1), Indoleamine 2,3-dioxygenase 2 (IDO-2), and Tryptophan 2,3-dioxygenase (TDO) [22, 23]. Among these, IDO-1 is the most extensively studied in the context of HIV due to its strong inducibility by pro-inflammatory cytokines such as interferon-γ (IFN-γ), and its expression in key immune cells including dendritic cells and macrophages. IDO-2, in contrast, exhibits markedly lower catalytic efficiency and is less responsive to inflammatory stimuli; its biological role remains less clearly understood [23]. TDO, on the other hand, is constitutively expressed in the liver and primarily regulated by systemic Trp concentrations and glucocorticoids [24]. As a result, TDO is more associated with the homeostatic regulation of Trp levels rather than with immune-driven responses. The Kyn pathway stands at the intersection of immunity and metabolism, as IDO-1 is the enzyme part of the rate-limiting step for Trp to Kyn as stimulated by proinflammatory cytokines. Because IDO and TDO catalyze the conversion of Trp into N-formylkynurenine, which is rapidly converted to Kyn, this initial step determines the amount of Trp that enters the Kyn-Trp pathway. As such, it regulates the overall rate at which downstream Kyn metabolites are produced, thereby justifying their designation as the rate-limiting enzymes of the Trp–Kyn pathway [25]. This is crucial in controlling both acute and chronic infections while impacting inflammation [16]. Additionally, the Trp–Kyn pathway produces downstream by-products such as quinolinic acid (QUIN) and 3-hydroxykynurenine (3-HK), which are known to be neurotoxic at elevated concentrations [26–28]. The upregulation of these metabolites has been associated with dysregulated inflammation, sustained immune activation, and metabolic disruption-all of which have been linked to neuronal damage [26–28].

There is a link between increased immunological dysfunction and the excessive Trp–Kyn metabolism that happens during primary HIV-1 infection, and this is characterized by a decrease in Trp and elevation of Kyn levels within PLWH [29, 30]. The elevated levels of Kyn in PLWH are known to correlate with higher levels of immune activation markers, inclduing neopterin and/or IFN- γ [26], which upregulate the activity of rate-limiting enzyme IDO-1, the enzyme that converts Trp to Kyn [26, 28].

The Trp–Kyn pathway is one of the most extensively investigated pathways in HIV-1-related studies [28, 31]. However, to the best of our knowledge, no comprehensive review currently provides an overview of this topic in a context-specific manner. Furthermore, there is no clear consensus on which specific Trp–Kyn metabolites in the peripheral blood of PLWH  are most frequently studied and consistently linked to HIV-1 infection. Therefore, this systematic review aims to explore the peripheral metabolic profiles of the Trp–Kyn pathway metabolites in PLWH. The objectives are to (1) identify the most frequently investigated metabolites within this pathway and (2) determine whether these metabolites exhibit consistent trends in their levels during HIV-1 infection. The findings from this study may provide researchers with a concise overview of all related studies in this field, eliminating the need to navigate the extensive body of literature. Additionally, these insights may help identify potential targets for therapeutic, diagnostic, and prognostic applications.

## Methods

### Study design

This was a narrative systematic review aimed at summarizing the extent of literature on the peripheral profiles of the Trp–Kyn pathway in PLWH. The study has been done according to PRISMA guidelines [32] and the reporting of the study conforms to broad EQUATOR guidelines [33]. The protocol has been registered in PROSPERO under the ID CRD420250564274. This study has been approved by the North‐West University Health Research Ethics Committee (NWU‐HREC): NWU-00108-25-S1.

### Eligibility criteria

Clinical studies were included that investigated the Trp–Kyn pathway in adults (> 18 years) diagnosed with HIV (i.e., HIV-positive) who received ART treatment. No restrictions regarding ART medication types or treatment duration were made. Studies were included only if participants were on antiretroviral therapy (ART), in alignment with current global treatment guidelines recommending immediate initiation of ART upon diagnosis [34]. This criterion was applied to ensure clinical relevance and to reflect the metabolic profiles of the majority of PLWH in the modern treatment era. All studies were required to investigate metabolites of the Trp–Kyn pathway and measure them from peripheral blood samples, specifically serum or plasma. All other sample types (e.g., cerebrospinal fluid) were excluded, as they fell outside the scope of this review. This review focused on investigating peripheral Trp–Kyn pathway metabolite levels to aid in identifying key markers that may be explored for their diagnostic or prognostic potential. All metabolomic techniques/platforms were considered for inclusion (i.e. chromatography-based techniques, coupled with mass spectrometry). To qualify as a control group or comparative arm, studies needed to include HIV-1-negative controls. Exclusion criteria included pre-clinical studies, such as those using animal or cell culture models, as well as review articles. Studies involving co-infected participants, pregnant individuals, or those undergoing treatments including  cancer therapies, tuberculosis treatment, or interventions targeting neurological, psychiatric, or cognitive disorders, were excluded, as these conditions and treatments are known to influence Trp–Kyn metabolic profiles [35, 36]. This exclusion was employed to determine whether alterations in the pathway are directly attributable to HIV and its treatment, rather than to other co-existing conditions. Additionally, studies were excluded based on lifestyle factors including if more than 25% of participants were smokers, consumers of alcohol, or drug users. To maintain the scientific rigor of this review, only indexed, peer-reviewed articles were considered, with no inclusion of grey literature. Furthermore, in health-related research, it is well-established that excluding grey literature does not significantly influence the overall findings of related systematic reviews [37].

### Data sources

We electronically searched for publications in PubMed, Scopus, and Web of Science databases based on all studies published until 25 September 2024. Eligible studies included only published studies in English, as the exclusion of non-English studies does not significantly alter the overall findings of health-related reviews [38]. The search strategy was executed without publication date limitations. The full search criteria for each database are included in Supplementary File 1. The following search terms were applied to PubMed: (HIV [tw] OR HIV [mh] OR "aquired immunodeficiency syndrome" [tw] OR AIDS [mh]) AND (tryptophan [tw] OR tryptophan [mh] OR kynurenine [tw] OR kynurenine [mh] OR quinolinic acid [tw] OR quinolinic acid [mh] OR Kynurenic acid [tw] OR kynurenic acid [mh] OR 3-hydroxykynurenine [tw] OR 3-Hydroxyanthranilic Acid [mh] OR picolinic acid [mh] OR picolinic acid [tw] OR anthranilic acid [mh] OR NAD [mh] or Xanthurenic acid [mh]).

**Fig. 1:**
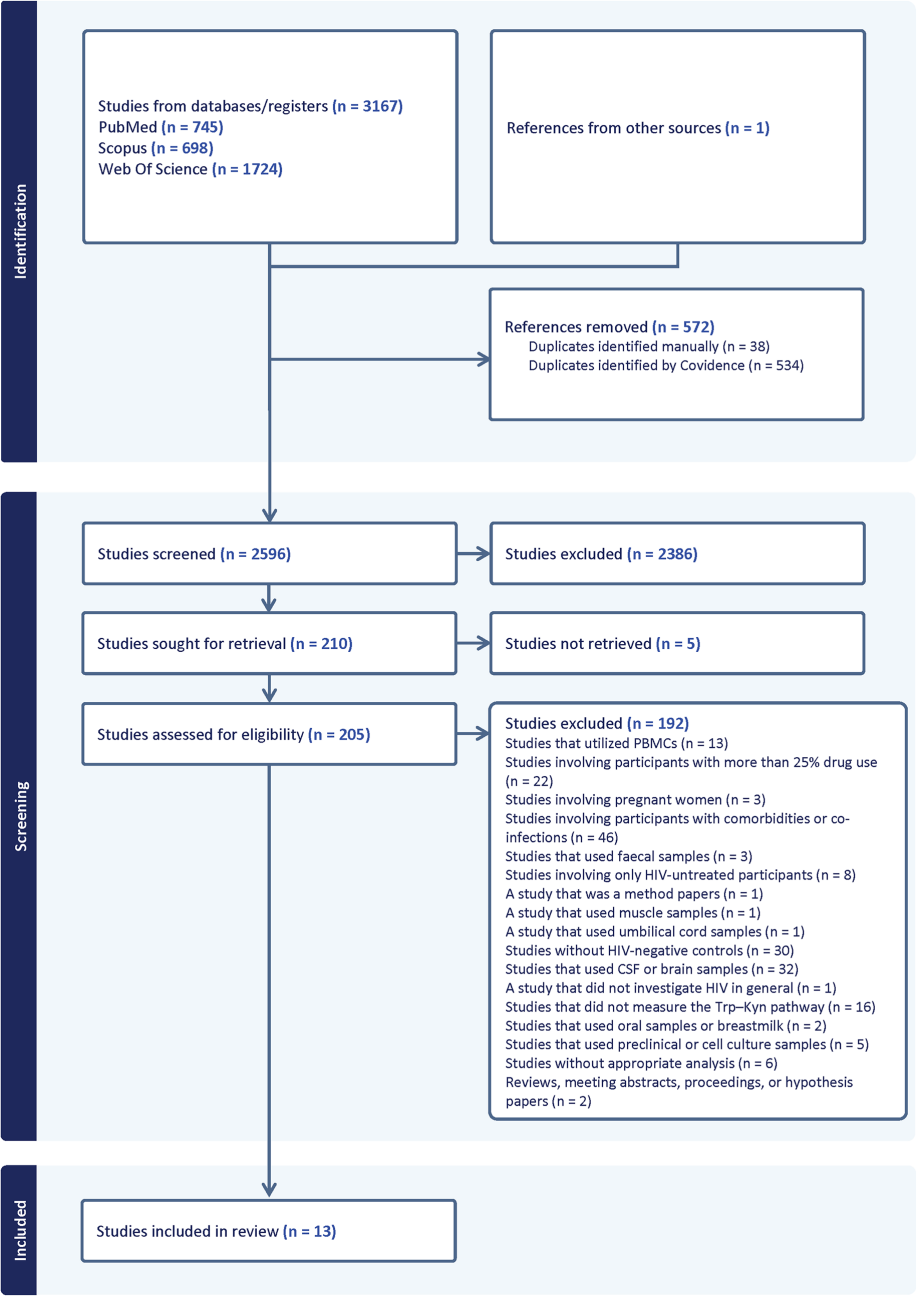
PRIMSA flow diagram

In addition, we also (1) reviewed reference sections of eligible articles and manually searched for relevant publications and (2) consulted with the contact authors of the included studies. This search strategy and the retrieved articles are shown in Fig. 1.

### Data selection

All articles were retrieved and loaded onto a single database using a reference manager, Covidence AI. Two of the co-authors (TS and LKA) independently identified studies meeting the inclusion criteria. Where there was a discrepancy in article inclusion/exclusion, this was discussed amongst all authors, and a decision was made regarding its suitability.

### Quality assessment of included studies

The quality of the included studies was assessed using Kappa statistics and the Joanna Briggs Institute (JBI) critical appraisal tools. We adapted the JBI tools by incorporating a Likert scale [39] to provide a quantitative measure of study quality as conducted previously [40–43]. For the evaluation, we focused on the JBI quality questions from the Checklist for Analytical Cross-Sectional Studies that could influence the findings. These questions addressed, (1) the definition and description of cohort information, (2) the validity and conditions of measurement (e.g., viral load and CD4 + count), and (3) the consideration of confounding factors and statistical analysis.

Specifically, we assessed the studies with the following questions:

1. Were the criteria for sample inclusion clearly defined, and were the study subjects and setting described?
2. Was the method for investigating clinical outcomes objective, valid, and reliable?
3. Did the study consider confounding factors and employ appropriate statistical analysis?

Each question was rated as follows: ‘0 = no’, ‘1 = partly’, or ‘2 = yes’. Studies that answered all the questions with a total rating of ≥ 5 were classified as high quality. Those with a rating between 3 and 4 were considered intermediate quality, and those with a rating of ≤ 2 were classified as low quality (see Supplementary Table 1).

### Potential confounders

Several factors may influence the findings reported in the reviewed studies measuring the metabolic profile. Among several potential confounders, we considered key factors such as: viral load [44], CD4 + count [45], sex [46], and subtype/geographical region [47]. We therefore investigated whether these confounders potentially influenced metabolic profiles and the findings reported in the reviewed studies. To address this, we first stratified participants based on viral load. Viral suppression was defined as having less than 2.6 log (50) copies/ml of HIV in the blood [48–50], while viral loads greater than 2.6 log (50) copies/ml were classified as non-suppressed. All studies reported viral load as a group mean (standard deviation) (Table 1). Secondly, we stratified the studies according to mean or median CD4 + counts of less than or greater than 200 cells/μL. Mean values were primarily used for stratification; however, median values were utilized in instances where mean values were unavailable. Lastly, we aimed to assess whether variations in sex and HIV-1 subtype could influence the metabolite levels in the blood (plasma and/or serum) of PLWH. This consideration is important because variations in sex, HIV-1 subtypes, and subtype-specific viral protein amino acid sequences can affect Trp–Kyn metabolite levels [51].

**Table 1:** Study characteristics for the included studies

Reference	Number of Participants	Geographical location/ HIV-1 subtype	CD4 count (cells/μL)	Viral loads (copies/ml)	Age (years)	Gender n (%)	Treatment Type
Akusjärvi et al. (2023)	People living with HIV (PLWH): n = 29 HIV negative controls: n = 37	Stockholm, Sweden	PLWH: Not declared (N/D) HIV negative controls = N/D Nadir CD4 PLWH: N/D HIV negative controls = N/D	N/D	PLWH = N/D HIV negative controls = N/D	PLWH N/D HIV negative controls N/D	Antiretroviral therapy (ART) Regimen: N/D
[52]	PLWH: n = 22 HIV negative controls: n = 22	India	PLWH: 624 (524–746) HIV negative controls: N/D Nadir PLWH = 229 (176–318) HIV-negative controls = N/D	< 150 (100%)	PLWH: 45 (43–48) HIV negative controls: 45 (43–47)	PLWH Male: n = 13 (59%) Female: n = 9 (41%) HIV-negative controls Male: n = 12 (55%) Female: n = 10 (45%)	ART Regimen NRTI + NNRTI-based regimen AZT/3TC/NVP: n = 13 (59%) TDF/3TC/EFV: n = 9 (41%)
Baer et al. (2021)	PLWH: n = 205 HIV negative controls: n = 99	United States of America (USA)	PLWH: 512 (30–2135) HIV negative controls: 859 (360- 1717) Nadir CD4 PLWH: 143 (0–668) Control: 581 (242- 1198)	N/D	PLWH: 52 (35–87) HIV negative controls: 52 (35–83)	PLWH Male: n = 166 (81%) Female: n = 39 (19%) HIV-negative controls Male: n = 80 (81%) Female: n = 10 (19%)	ART Regimen: N/D
[53]	PLWH: n = 76 HIV negative controls: n = 16	Shanghai, China	PLWH = 248 (121–297) HIV negative controls = N/D Nadir CD4 PLWH = (N/D) HIV negative controls = N/D	< 40 (97%)	PLWH = 32 (28–39.5) HIV negative controls = 32 (27–47)	PLWH Male: n = 58 (76%) Female: n = 18 (24%) HIV negative controls Male: n = 10 (62%) Female: n = 6 (38%)	ART Regimen: 2 NRTIs + 1 NNRTIs: n = 73 (96.1%) 2 NRTIs + 1 PIs: n = 3 (3.9%)
[54]	PLWH: n = 127 HIV negative controls: n = 25	Shanghai, China	PLWH = 403 (332–560) HIV negative controls = N/D Nadir CD4 PLWH = N/D HIV negative controls = N/D	PLWH = 40 738 (14 125–112 202)	PLWH = 32 (27–44) HIV negative controls = N/D	PLWH Male: n = 117 (92.1%) Female: n = 10 (7.9%) HIV negative controls: N/D	ART Regimen: NRTI + NNRTI-based regimen TDF + 3TC + EFV: n = 116 (91.3%) ZDV + 3TC + EFV/NVP: n = 7 (5.5%) TDF + 3TC + RAL: n = 2 (1.6%) TDF + 3TC + LPV/r: n = 2 (1.6%)
[8]	PLWH: n = 50 HIV negative controls: n = 50	Cape town, South Africa	PLWH = 517 (399–716.5) HIV negative controls = N/D Nadir CD4 PLWH = N/D HIV negative controls = N/D	PLWH = 20 (10–53)	PLWH = 36 (33–45) HIV negative control = 35 (31–42)	PLWH Male: n = 15 (30%) Females: n = 35 (70%) HIV negative controls Males: n = 16 (32%) Females: n = 33 (68%)	ART: Regimen: NRTI + NNRTI-based regimen EFV 600 mg + TDF DF 300 mg + emtricitabine 200 mg: n = 46 (92%) NVP 200 mg /lamivudine 150mg + ZDV 300 mg (Zidovudine n = 1 (2%) Lamivudine/EFV/Abacavir: n = 1 (2%) Ritonavir 50 mg + lopinavir 200 mg (Aluvia)/ TDF /Lamivudine: n = 1 (2%)
[55]	PLWH: n = 42 HIV negative controls: n = N/D	N/D	PLWH = N/D HIV negative controls = N/D Nadir CD4 PLWH = (N/D) HIV negative controls = N/D	N/D	PLWH = 37.5 (21–54) HIV negative controls = N/D	PLWH Male: n = 40 (95.2%) Females: n = 2 (4.8%) HIV negative controls = N/D	ART (NRTI) Regimen: Zidovudine (azidothymidine, AZT)
[56]	PLWH: n = 88 HIV-negative controls: n = 50	Canada	PLWH = 531.3 (± 267.4) HIV negative controls = 812.6 (± 273) Nadir CD4 PLWH = N/D HIV negative controls = N/D	< 40 (100%)	PLWH = 48.4 (± 9) HIV negative controls = 45.0 (± 9.9)	PLWH Male: n = 73 (83%) Female: n = 15 (17%) HIV negative controls Male: n = 35 (70%) Female: n = 15 (30%)	ART Regimen: N/D
[57]	PLWH: n = 19 HIV negative controls: n = 18	China	PLWH: 399 (78–765) HIV negative controls = N/D Nadir CD4 PLWH = (N/D) HIV negative controls = N/D	19.95 (19.95–129.15)	PLWH = 35 (23–74) HIV negative controls = 39 (21–47)	PLWH Male: n = 19 (100%) Female: n = 0 (0%) HIV negative controls Male: n = 18 (100%) Female: n = 0 (0%)	ART Regimen: LPV/r
[58]	PLWH: n = 100 HIV negative controls: n = 21	Pretoria, South Africa	PLWH = 413.33 HIV negative controls = N/D Nadir CD4 PLWH = N/D HIV negative controls = N/D	N/D	PLWH = 35.79 (25–49) HIV negative controls = 31.45 (25–52)	PLWH Male: n = 69 (69%) Female: n = 31 (31%) HIV negative controls Male: n = 17 (80.9%) Female: n = 4 (19%)	ART Regimen: N/D
[59]	PLWH: n = 39 CD4^+^ < 350 = 24 CD4^+^ > 500 = 15 HIV negative controls: n = 19	San Francisco USA	PLWH: N/D CD4^+^ < 350 = 229 (155–279) CD4^+^ > 500 = 628 (532–1011) HIV-negative control = 628 (532–1011) Nadir CD4 CD4^+^ < 350 = 21 (7–56) CD4^+^ > 500 = 105 (49–311) HIV negative controls = N/D	Undetectable (100%)	PLWH: N/D CD4^+^ < 350 = 47 (43–52) CD4^+^ > 500 = 49 (47–55) HIV negative controls = 47 (37–55)	PLWH Male: n = 38 (97%) Female: n = 1 (3%) HIV negative controls Male: n = 16 (84.2%) Female: n = 3 (15.7%)	ART Regimen: N/D
[60]	PLWH: n = 38 HIV negative controls: n = 18	Shanghai, China	PLWH = 357 (264–527) HIV negative controls = N/D Nadir CD4 PLWH = N/D HIV negative controls = N/D	N/D	PLWH = 32 (26–50) HIV negative controls = 31 (26–41)	PLWH Male: n = 34 (89.4%) Female: n = 4 (10.5%) HIV negative controls Male: n = 12 (66.6%) Female: n = 6 (33.3%)	ART Regimen: TDF + 3TC + EFV: n = 32 (84.2%) TDF + 3TC + LPV/r: n = 2 (5.3%) AZT + 3TC + EFV: n = 3 (7.9%) TDF + 3TC + RAL: n = 1 (2.6%)
[61]	PLWH: n = 30 HIV negative controls: n = 40	Guangzhou, China	PLWH = 459.1 ± 153.15 HIV negative controls = N/D Nadir CD4 PLWH = N/D HIV negative controls = N/D	PLWH = < 20 (100%)	PLWH = 37.63 (± 9.23) HIV negative controls = 32.53 (± 5.51)	PLWH Male: n = 27 (90%) Female: n = 3 (10%) HIV negative controls Male: n = 28 (70%) Female: n = 12 (30%)	ART Regimen: TDF + 3TC + EFV: n = 29 (96.7%) AZT/3TC + EFV: n = 1 (3.3%)

*ART* antiretroviral therapy, *AZT* Zidovudine, *DDI* didanosine, *DDC* 2′3′-dideoxycytosine, *EFV* efavirenz, *N/D* not declared, *NRTIs* nucleoside reverse transcriptase inhibitors, *NNRTIs* non-nucleoside reverse transcriptase inhibitors, *NVP* Nevirapine, *PIs* protease inhibitors, *PV/r* lopinavir/ritonavir, *RAL* raltegravir, *SMX* sulfamethoxazole, *TDF* tenofovir, *TMP* trimethoprim, *ZDV* zidovudine, *3TC* lamivudine

In certain studies, CD4 counts are reported without units of measurement, as the original studies did not specify them. Data is reported as median (range) or mean (SD). Where studies reported both the city and country, these were recorded accordingly. Certain studies only reported the country, and in such cases, only the country was recorded

## Results

### Study characteristics

Using this search strategy, a total of n = 3,167 studies were identified. One article was added through reference scanning. After removing n = 572 duplicates, a total of n = 2,596 article abstracts and titles were screened. Of these, n = 2,386 articles were excluded. Subsequently, n = 210 articles were sought, although n = 5 were not retrievable. Therefore, full-text analysis was conducted for n = 205 articles to assess eligibility, resulting in the exclusion of n = 192 articles for various reasons listed in Fig. 1. Ultimately, n = 13 studies were included for review and data extraction (Fig. 1).

Cohort information was extracted from all articles. Most of the studies included were cross-sectional in design, with only one utilizing a longitudinal design. For the longitudinal study, data was extracted exclusively from the period when participants were treatment-experienced.

Across all studies included, the study participants included n = 865 PLWH and n = 327 HIV-negative controls. One study did not report the number of HIV-negative controls [55]. The mean participant numbers were n = 67 PLWH and n = 32 HIV-negative controls per study. The majority of the studies reported the ages of both PLWH (11/13, 85%) and HIV-negative controls (10/13, 77%), with participants' ages ranging from 21 to 87 years. Regarding sex distribution, the majority of studies provided this information for PLWH and HIV-negative controls, except for three studies, which did not report sex for both PLWH and/or HIV-negative controls [54, 55, 62]. Across all included studies, men represented the majority of PLWH (669/865, 77%), as well as the HIV-negative controls (216/327, 66%). As part of the inclusion criteria, all PLWH had to be treatment-experienced. However, not all of the studies have reported the exact treatment regimen, with 9/13 (69%) indicating the regimen. There were varied levels of ART regimens across all the studies (Table 1), and the majority of the participants were receiving first-line regimens of Nucleoside Reverse Transcriptase Inhibitor (NRTI) and Non-Nucleoside Reverse Transcriptase Inhibitor (NNRTI). The majority of studies have not reported the duration of treatment; therefore, this variable could not be evaluated in our analysis.

### Quality of assessment of included studies

Between the independent raters, the Kappa score was 0.53, indicating moderate agreement. Approximately 54% of the studies were classified as having a high quality, and the remaining studies were considered of intermediate quality, with no studies rated as low quality (Supplementary Table 1). Based on these findings, several recommendations are made in the latter part of the review.

### Sample matrix, chromatography and metabolomics tools utilized

As part of the inclusion criteria, all investigations were to be done in peripheral blood sample types (plasma and/or serum). The majority of the studies made use of plasma samples (9/13, 69%) (Table 2). Several metabolomic analytical platforms were used to measure metabolites of the Trp–Kyn pathway. The majority of the studies used Ultra Performance Liquid Chromatography (UPLC)/Mass Spectrometry (MS) (3/13, 23%), followed by LC/MS (2/13, 15%), and at least one study (1/13, 8%) employed High-Performance Liquid Chromatography (HPLC)/MS/MS, HPLC-fluorescence, Gas Chromatography (GC)/MS, Liquid Chromatography-Multiple Reaction Monitoring (LC-MRM)/MS, Solid-Phase Extraction (SPE)-LC–MS/MS or Ultra-High-Performance Liquid Chromatography (UHPLC)/MS/MS. One study utilized both UPLC/MS and GC/MS (1/13, 8%). Another study reported the chromatography technique (HPLC) but did not specify the detection method (1/13, 8%).

**Table 2:** Tryptophan–Kynurenine pathway metabolite levels in people living with HIV

Reference	Sample type	Tryptophan–Kynurenine metabolite/s investigated	Metabolomics parameters	Key findings	Other key findings
Akusjärvi et al. (2023)	Plasma	Targeted analysis: Tryptophan (Trp) and Kynurenine (Kyn)	Liquid chromatography/mass spectrometry (LC/MS) Column: XBridge Peptide BEH C18 column Mobile phases: A—(20 mM Ammonium hydroxide) (NH_4_OH) B- (20 mM NH_4_OH in Acetonitrile (ACN), pH 10.0) Protein precipitation: 20 mM NH_4_OH	The levels of Kyn and Trp were decreased in people living with HIV (PLWH) compared to HIV negative controls (adjusted *p* < 0.05)	Between PLWH and HIV negative controls there were 19 metabolites that showed a significant difference (adjusted *p* < 0.05) There were 12 inflammatory markers that were significantly higher in PLWH than HIV negative controls (adjusted *p* < 0.05)
[52]	Plasma	Untargeted analysis: Trp) Kyn/Trp ratio	Ultra-high-performance liquid chromatography/mass spectrometry/mass spectrometry (UHPLC/MS/MS) Column: N/D Mobile phase: N/D Protein precipitation: Methanol (MeOH)	PLWH had significantly lower levels of Trp compared to HIV-negative controls (*p* = 0.002) PLWH had a significantly higher Kyn/Trp ratio as compared to the HIV negative controls (*p* = 0.006) There were no significant differences in Kyn levels between PLWH and HIV negative controls	A total of 250 metabolites differed significantly (*p* < 0.05, q < 0.1) between PLWH and HIV-negative controls Plasma levels of several essential amino acids except for histidine, branched-chain amino acids, and aromatic amino acids (phenylalanine, tyrosine, trp) were significantly lower in PLHIV compared to HIV-negative controls
Baer et al. (2021)	Serum	Targeted analysis: Indoleamine-2,3-dioxygenase (IDO) (Kyn/Trp ratio)	High-performance liquid chromatography (HPLC) (detection method not defined) Column: N/D Mobile phase: N/D Protein precipitation: N/D	PLWH had a higher Kyn/Trp ratio compared to the HIV negative controls (*p* < 0.0001) which was indicative of higher IDO activity	The Kyn/Trp ratio values of younger PLWH (< 50 years old) were higher than older (> 50 years old) HIV negative controls (median (min, max): 0.047 (0.016, 0.124) vs. 0.065 (0.025, 0.361) Age was linked to a significantly increased Kyn/Trp ratio (*p* < 0.0001) and the increase was independent of HIV infection status (*p* = 0.92, r^2^ = 0.18 for the interaction) The Kyn/Trp ratio showed no differences based on gender, race, or ethnicity There was no correlation between the Kyn/Trp ratio and either the current CD4^+^ or nadir CD4^+^ cell counts PLWH showed a decrease in Kyn/Trp ratio as plasma lipopolysaccharide (LPS) increased, in contrast to HIV negative controls, who showed no correlation between Kyn/Trp ratio and LPS (*p* = 0.0071) Neopterin and the Kyn/Trp ratio were positively correlated in both groups (PLWH and HIV negative controls) (*p* < 0.0001)
[53]	Plasma	Targeted analysis: Trp, Kyn and IDO activity (Kyn/Trp ratio)	Ultra-performance liquid chromatography (UPLC)/MS Column: Agilent Hypersil ODS Mobile phases: Acetate buffer and ACN Protein precipitation: Perchloric acid	There were no significant differences in Trp and Kyn concentrations between PLWH and HIV-negative controls (*p* > 0.05) IDO activity (Kyn/Trp ratio) was significantly higher in PLWH compared to HIV negative controls (*p* = 0.0148)	Plasma sCD14 levels did not correlate with Kyn, Trp, or IDO with PLWH or HIV negative controls
[54]	Plasma	Targeted: IDO activity (Kyn/Trp ratio)	UPLC/MS Column: Poroshell 120 EC-C18 Mobile phases: Ammonium formate and formic acid (HCO_2_H) in water (H_2_O) as well as ACN Protein precipitation: N/D	IDO activity was significantly higher in PLWH compared to HIV-negative controls (*p* < 0.05)	There was a significant correlation (r = 0.36, *p* < .0001) between the HIV DNA and IDO activity
[8]	Plasma	Targeted: Kyn, L-Trp, 3-Hydroxykynurenine, 5-Hydroxyindoleacetic acid, Kynurenic acid, Indole-3-acetaldehyd, Xanthurenic acid, Indole-3-acetate and Quinoldic acid: 2-quinoline carboxylic acid, IDO	Liquid chromatography-multiple reaction monitoring/mass spectrometry (LC-MRM/MS) Column: Kinetex C18 column Mobile phases; formic acid in H_2_O / ACN and Ammonium fluoride in water/CAN Protein precipitation: MeOH –ethanol mixture	Trp (*p* < 0.01) and Xanthurenic acid (*p* < 0.05) was significantly lower in PLWH compared to HIV negative controls Trp/Kyn ratio (*p* < 0.05) and 3-Hydroxykynurenine (*p* < 0.05) was significantly higher in PLWH compared to HIV-negative controls No significant differences were reported for the other Trp–Kyn pathway metabolites including 5-Hydroxyindoleacetic acid, Indole-3-acetaldehyde, Kynurenic acid, Indole-3-acetate and Quinoldic acid: 2-quinoline carboxylic acid	There was a correlation between the Kyn/Trp ratio ratio and CD4 count in PLWH (*p* < 0.001) There was also a significant correlation between tryptophan and CD4 count in PLWH (*p* < 0.05) A significant correlation was observed between viral load and tryptophan (*p* < 0.05), 3-hydroxykynurenine (*p* < 0.05), 5-hydroxyindoleacetate (*p* < 0.01), the Kyn/Trp ratio (*p* < 0.0001), and Kyn (*p* < 0.01) in PLWH
[55]	Serum	Targeted: Trp & Kyn	HPLC- fluorescence Column: N/A Mobile phases: N/D Protein precipitation: N/D	The serum concentration of Kyn was significantly higher in PLWH compared to HIV negative controls (*p* < 0.01) Trp levels on the other hand were significantly lower in PLWH compared to HIV negative controls (*p* < 0.01)	Serum neopterin showed a strong positive correlation with Kyn and Kyn/Trp ratio (r = 0.842, *p* < 0.001) in PLWH IFN-γ concentrations were significantly elevated in PLWH compared to HIV negative controls (*p* < 0.01)
[56]	Plasma	Targeted: Kyn and Kyn/Trp ratio (IDO activity)	Solid phase extraction (SPE)-LC–MS/MS Colum: Atlantis dC18 column Mobile phases: HCO_2_H in H_2_O and ACN Protein precipitation: N/D	PLWH had higher Kyn levels compared to HIV-negative controls (*p* = 0.0079) IDO activity was significantly higher in PLWH compared to HIV-negative controls (*p* = 0.0020)	When comparing elite controllers to PLWH that are successfully treated with ART and HIV negative controls, similar levels of Trp catabolites and IDO activity were maintained IDO activity was slightly elevated between elite controllers and HIV negative controls (*p* = 0.0072)
[57]	Plasma	Untargeted metabolomics: Kyn	UPLC/MS and gas chromatography-mass spectrometry (GC/MS) Column: The GC column contained 5% phenyl, with temperature slope ranging from 40 to 300 °C in 16 min Mobile phases: LC and GC: N/D Protein precipitation: LC and GC: N/D	There was no significant difference in Kyn levels between PLWH and HIV negative controls (*p* = 0.30)	A 67-metabolite signature mapping to trp, histidine, acyl carnitine, ketone bodies, and fatty acid metabolism distinguished PLWH from HIV-negative controls Nineteen out of the 67 altered metabolites, including histidine, kynurenine, and 3-hydroxybutyrate (BHBA), recovered after ART Histidine was positively correlated with the presence of CD4 + T lymphocytes Butyrylcarnitine and myristic acid from plasma in treatment-naïve patients predicted dyslipidemia caused by ART with 87% accuracy
[58]	Serum	Targeted: Trp	GC/MS Column: Phenomenex ZB-AAA column Carrier gases: N/D Protein precipitation: EZ:faast™ kit (Phenomenex) but solvent not reported	PLWH had significantly lower levels of Trp compared to HIV-negative controls (Fold change 0.49, *p* = 0.01)	PLWH had up-regulation of aspartic acid, phenylalanine and glutamic acid compared to HIV-negative controls Trp and tyrosine were down-regulated whereas cystine levels were higher in PLWH and treatment naïve PLWH as compared to both HIV treated and HIV-negative controls
[59]	Plasma	Targeted: IDO	LC/MS Column: Synergi Polar RP Mobile phases: ACN, MeOH and HCO_2_H Protein precipitation: trifluoroacetic acid	All PLWH had higher IDO-1 activity compared to HIV negative controls (*p* $\le 0.04$)	Increased mucosal apoptosis was positively correlated with higher plasma sCD14 levels (rho = 0.46, *p* = 0.04) and the Kyn/Trp ratio (rho = 0.42, *p* = 0.08) The median levels of soluble monocyte activation makers and LPS receptor, sCD14 were higher in PLWH than HIV negative controls (2.7 vs. 1.8mg/ml; *p* = 0.01)
[60]	Plasma	Targeted: Trp, Kyn, and IDO	UPLC/MS Column: Poroshell 120 EC-C18 Mobile phases: Ammonium formate and HCO_2_H in water as well as ACN Protein precipitation: N/D	Trp was significant lower in PLWH compared to HIV-negative control (< 0.05) No significant differences were reported for Kyn and IDO activity (p > 0.05)	Pre-ART, PLWH demonstrated notably elevated plasma concentrations of soluble intercellular adhesion molecule 1 (sICAM-1), soluble vascular cell adhesion molecule 1(sVCAM-1), and IDO activity in comparison HIV-negative controls Post-ART, both IDO activity and sICAM-1 levels experienced a significant decrease, with IDO activity reaching levels comparable to those observed in HIV-negative controls
[61]	Serum	Targeted: Trp, Kyn, and IDO	HPLC–MS/MS Column: C18 analytical column Mobile phases: HCO_2_H in water and HCO_2_H in MeOH Protein precipitation: MeOH	In PLWH, Kyn was significantly higher compared to HIV negative controls (*p* < 0.01) In PLWH, Trp was significantly lower compared to HIV negative controls (*p* < 0.01) IDO-1 activity (Kyn/Trp ratio) was significantly higher in PLWH compared to HIV negative controls (*p* < 0.001)	Homeostasis model assessment for insulin resistance was positively correlated with Kyn/Trp (0.33), Kyn (0.33), and PAGln (0.31), whereas a negative correlation with 5‐HT (− 0.30)

*UHPLC/MS/MS* Ultra-high-performance liquid chromatography/mass spectrometry/mass spectrometry, *PLWH* People Living with HIV, *Trp* Tryptophan, *Kyn/Trp ratio* Kynurenine to Tryptophan ratio, *Kyn* Kynurenine, *HPLC* High-performance liquid chromatography, *IDO* Indoleamine 2,3-dioxygenase, *UPLC/MS* Ultra-performance liquid chromatography/mass spectrometry, *sCD14* Soluble CD14, *LC-MRM/MS* Liquid chromatography-multiple reaction monitoring/mass spectrometry, *GC/MS* Gas chromatography-mass spectrometry, *SPE-LC–MS/MS* Solid-phase extraction-liquid chromatography-tandem mass spectrometry, *ART* Antiretroviral therapy, *IFN-γ* Interferon-gamma, *LPS* Lipopolysaccharide, *N/D* Not declared

Further, the type of column, mobile/gas phases, and solvents used for protein precipitation may potentially influence the quantification of Trp-Kyn metabolites. The column is inherently determined by the method of metabolomics utilized. Of all the included studies, 9/13 (69%) reported the type of column used in the analysis. The majority of these studies utilized reversed phase (RP) columns (6/9, 67%) including the C18 Analytical Column, Poroshell 120 EC-C18, Kinetex C18, XBridge Peptide BEH C18 column, and Agilent Hypersil ODS. The remaining studies utilized a Polar-Embedded Reversed-Phase LC Column (Synergi Polar RP), an LC Column (Atlantis dC18) and one study utilized a GC column (Phenomenex ZB-AAA) (Table 2). With regard to reporting of the mobile/gases phases, 8/13 (62%) reported this information. From these studies, the majority (7/8, 88%) utilized acetonitrile (ACN) as a mobile phase in liquid chromatography, either alone or in combination with other solvents such as water, methanol, or additives like formic acid and ammonium salts (Table 2). Lastly, in the studies reviewed, 7/13 (54%) reported the solvents used for protein precipitation. The majority of these studies utilized methanol, either alone or in combination with other solvents such as ethanol, ammonium hydroxide, or Tris (2-carboxyethyl) phosphine hydrochloride (TCEP) (Table 2).

### Trp–Kyn metabolites and HIV-1 infection

Several metabolites were investigated across the studies included in this review. The majority (11/13, 85%) of studies utilized targeted metabolomic approaches, whereas two employed untargeted metabolomics [52, 63]. For the targeted investigations, the majority of studies investigated the IDO activity (Kyn/Trp ratio) (n = 8/11, 73%), Trp (n = 7/11, 64%), and Kyn (n = 7/11, 64%) (Table 2). Across all studies, IDO activity was the most widely investigated (9/13, 69%).

Certain metabolites were investigated more frequently, inherently resulting in more supporting evidence. To account for this, we considered the frequency of investigations when contextualizing the findings. For this review, we applied a previously established criterion for identifying “noteworthy” metabolites/markers [41, 43, 64]. A metabolite was deemed noteworthy if it met the following conditions: (1) it was investigated in at least two independent studies (> 2), and (2) more than 75% of the studies reported a consistent trend in its levels in PLWH compared to HIV-negative controls. In other words, if over 75% of studies investigating a particular marker found consistent directional changes (lower, higher, no difference in PLWH) in its levels associated with HIV-1 infection, it was identified as a noteworthy candidate for future research.

**Fig. 2:**
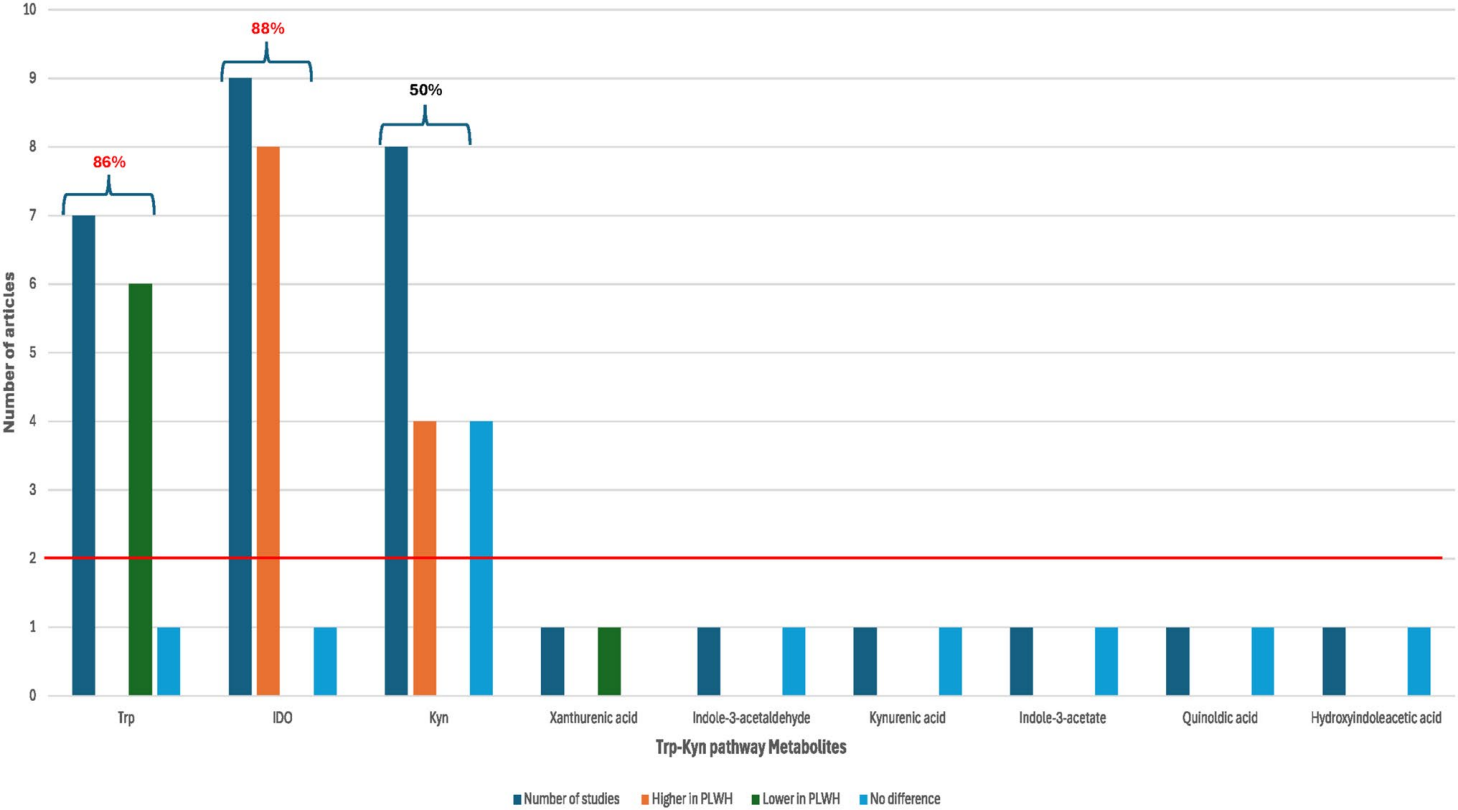
Frequency of metabolite investigation across all studies. The dark blue bars represent the number of studies that investigated each metabolite. Metabolites were considered noteworthy if they were investigated in more than two independent studies, as indicated by the red line cut-off (criterion one). The orange bars represent the number of studies reporting higher metabolite levels in PLWH, while the green bars represent studies reporting lower levels. The light blue bars indicate the number of studies that found no significant differences in metabolite levels between PLWH and HIV-negative controls

From all the markers investigated, Trp, Kyn, and IDO activity were studied in more than two independent studies, thus meeting the first criterion (Fig. 2, red cutoff line). Trp was consistently reported to be lower in PLWH, with 7 out of 8 studies (86%) supporting this finding. IDO activity was consistently higher in PLWH, with 8 out of 9 studies (88%) reporting this trend, thereby meeting the second criterion for noteworthy metabolites (Fig. 2). Interestingly, the findings for Kyn levels were not consistent. Among the studies, 4 out of the 8 studies (50%) reported higher Kyn levels in PLWH, while the remaining 4 out of 8 (50%) found no significant difference in Kyn levels between PLWH and HIV-negative controls. This lack of consistency prevented Kyn from meeting the criteria for a noteworthy marker.

### Confounders: viral load, CD4 + count, sex, age, and geographical region

Several potential confounders may have influenced the findings in the reported studies and, consequently, our interpretation. These factors warrant further acknowledgment.

Firstly, we aimed to determine whether viral load influenced the reported levels of Trp–Kyn metabolites in the included studies. The majority of the studies (n = 8/13, 62%) provided data on viral load. Among these, 7 out of 8 studies reported viral suppression. Therefore, there was no comparator arm (non-viral suppression) to facilitate a meaningful comparison. Nevertheless, there appears to be a consistent observation that Trp–Kyn metabolites remain elevated in PLWH despite viral suppression. Similarly, with CD4 count, most studies included participants with CD4 + counts > 200 cells/μL, limiting meaningful comparisons. Furthermore, none of the studies statistically adjusted for viral load. Despite this, there is a general agreement that even with higher CD4 + counts, certain metabolites remain dysregulated in PLWH compared to HIV-negative controls.

Next, we sought to determine whether skewed sex distribution within the studies influenced the reported metabolite levels. We observed that males represented the vast majority of both PLWH (669/865, 77%), as well as the HIV-negative controls (216/327, 66%). To explore whether sex influenced metabolite levels, we considered studies where females were the majority. In one such study [8], where females comprised the majority of participants, similar findings were reported. These included significantly lower Trp and Xanthurenic acid levels in PLWH compared to HIV-negative controls, along with significantly higher Kyn/Trp ratios and 3-HK levels in PLWH. While this suggests some consistency in the dysregulation of the Trp–Kyn pathway regardless of sex, the influence of sex on this pathway in PLWH cannot be definitively ascertained from the current data. Future investigations should prioritize analyses that address this factor. Further, only one study statistically adjusted for sex as a variable [65], whereas no studies have subdivided their population by sex when comparing metabolite levels.

Although determining the HIV-1 subtype is crucial for contextualizing findings, none of the studies included in this review reported the HIV-1 subtype. As a result, we were unable to assess whether subtype variation influenced the reported metabolite results. Instead, we explored whether geographical region could offer insights into differences in metabolite levels among PLWH. Interestingly, the majority of studies on this topic included participants from China (5/13, 38%), followed by the United States of America (USA) (2/14, 14%) and South Africa (2/14, 14%). Despite this geographical distribution, there did not appear to be a clear effect of geographic region on the direction of metabolite levels in PLWH compared to HIV-negative controls.

## Discussion

Several findings emerged from this systematic review study which include: 1) Trp, Kyn, and the Kyn/Trp ratio (indicative of IDO activity) were the most commonly investigated metabolites in this field of research; 2) evidence consistently demonstrated that Trp levels were lower in PLWH; while 3) IDO activity was consistently higher; and 4) despite the widespread investigation of Kyn, there was no clear consensus on its levels in PLWH, with some studies reporting higher levels and others finding no significant differences compared to HIV-negative controls.

The Trp–Kyn pathway has several key metabolites, including Kyn, kynurenic acid (KA), 3-HK, QUIN, Anthranilic acid (AA), Picolinic acid (PA), Nicotinamide (NAM), Nicotinamide mononucleotide (NMN) and Nicotinamide adenine dinucleotide (NAD +) [20, 66]. Several of these metabolites have been implicated in disease of several disorders, and this pathway has been widely investigated in PLWH. Despite the numerous metabolites in the kynurenine pathway, our review found that the literature on HIV-1 and PLWH is predominantly focused on Trp, Kyn, and the Kyn/Trp ratio, which is indicative of IDO-1 activity. While it is well-known that this pathway has been extensively studied, it was surprising to find that the majority of evidence specifically compares these metabolites between PLWH and HIV-negative controls. It is possible that other metabolites in this pathway, which were not highlighted in our review, are being investigated in relation to clinical outcomes in PLWH, rather than HIV-1 infection itself. For instance, metabolites like KA and QUIN have been widely implicated in the development of HAND [67, 68], as well as in the effects of increased age in the HIV-1 population [15]. However, this review highlights the need for further investigation into the levels of all metabolites within the Kyn pathway. Future studies should aim to determine whether the associations between these metabolites and comorbidities are driven or mediated by HIV-1 infection.

Trp catabolism primarily results in the production of Kyn through the enzymatic activity of IDO (IDO-1 &2) and TDO. While the Kyn/Trp ratio is commonly used as a surrogate marker of IDO activity, it is important to emphasize that this ratio serves as an indirect measure. It reflects the overall balance between Trp degradation and Kyn production rather than the specific activity of any single enzyme. Several studies have demonstrated a strong correlation between Kyn/Trp ratios and inflammatory mediators, including IFN-γ, interleukin-6 (IL-6), tumor necrosis factor-alpha (TNF-α), and neopterin [69, 70], supporting its relevance as an immunometabolic indicator. Therefore, measuring inflammatory mediators alongside Kyn/Trp levels provides important context for interpreting pathway activity (Table 2).

Further, based on this, the initial hypothesis for this pathway in PLWH is that Trp should consistently be lower, as it is catabolized in response to the inflammatory environment induced by HIV-1, including the production of cytokines like IFN-γ [29]. Higher inflammatory environments often stimulate greater IDO activity, leading to the expectation that IDO activity would be consistently higher in PLWH as highlighted in this review. This would, in turn, ensure that Kyn, as a result of Trp catabolism, is consistently higher in PLWH. However, we found Kyn levels to be inconsistent in PLWH compared to HIV-negative controls. While IDO and Trp levels may exhibit consistent patterns in PLWH due to chronic immune activation and inflammation, Kyn levels show more variability, possibly due to the complex regulation of the kynurenine pathway, which includes multiple enzymatic steps. The conversion of Trp to Kyn is not solely dependent on IDO. Other enzymes involved in the kynurenine pathway, such as kynurenine 3-monooxygenase (KMO) and kynurenine aminotransferase (KAT), can influence the flow of Trp metabolism and alter Kyn levels [71]. Additionally, the ART regimens used across the studies were varied, and this may have influenced the conversion of Trp to Kyn, potentially explaining the inconsistencies in Kyn levels in PLWH. Furthermore, other factors related to treatment, such as the duration of ART, may have also influenced these metabolic steps. Therefore, while Trp levels are consistently expected to be catabolized by IDO as part of the immune response induced by HIV-1, Kyn levels may be more widely regulated by additional factors, warranting further investigation. Moreover, individual Kyn measurements may be affected by study-specific variables such as population heterogeneity, sample collection timing, and analytical methodologies. In contrast, the Kyn/Trp ratio helps to normalize such variability and provides a more reliable reflection of IDO enzymatic activity and broader immunometabolic disturbances. Despite the inconsistent findings for Kyn alone, the consistent elevation in IDO activity across studies highlights a reproducible pattern of Trp–Kyn pathway dysregulation in PLWH. This consistency suggests that Trp depletion and IDO activity may serve as more robust and clinically relevant targets for the development of diagnostic, prognostic, and therapeutic strategies. This may also suggest that Trp and IDO levels could be more appropriate targets for the development of therapeutics, diagnostics, and prognostics.

Despite not considering factors like treatment regimen and treatment duration due to the vast variability across studies, we observed consistencies in the fact that, even though PLWH have low viremia and higher CD4 + counts, the metabolites of this pathway, particularly Trp and IDO, remain consistent in their levels in PLWH compared to HIV-negative controls. This is in line with previous findings suggesting that while cART may influence the pathway, it does not normalize it to levels seen in HIV-negative controls [53]. This is likely due to the chronic low-grade inflammatory profile experienced by PLWH on ART [72]. Therefore, targeting this pathway through therapeutic strategies, including cART, may offer additional benefits in mitigating inflammation and improving clinical outcomes for PLWH.

## Limitations and recommendations

Several limitations can be highlighted in this review. First, the limited number and heterogeneous nature of the included studies restricted our ability to conduct a meta-analysis. Second, the majority of studies were cross-sectional in design, providing only a snapshot of the metabolic profile of the cohort at a particular point in time. It would have been beneficial to include longitudinal studies, as these could provide insights into consistent trends in metabolite levels over time. Third, while we aimed to focus our review on specific metabolic platforms, it is important to note that different metabolomic tools were used to quantify the Trp–Kyn metabolites. These tools may have varied in terms of sensitivity, volatility, specificity, and reproducibility. Variations in solvents for protein precipitation, columns used for separation, and drying methods may also contribute to discrepancies in results across studies, and thus our findings should be interpreted in light of this. Fourth, the use of different sample types, such as serum and plasma, can also potentially affect the levels of Trp–Kyn metabolites [73]. Fifth, regarding the different HIV treatment regimens and durations, several studies did not disclose the specific antiretroviral regimens used by the cohorts. In cases where regimens were reported, they varied across studies, limiting our ability to assess how variations in treatment protocols might have influence Trp–Kyn metabolite levels. This lack of information obscures the impact of specific drug classes on metabolic pathways. Sixth, the lack of clarity in the study designs of some studies made it challenging to fully interpret the data. Future studies in this area should develop uniform standards for reporting study design. To improve the reliability and comparability of future research, we recommend conducting longitudinal studies with consistent methodologies and standardized protocols for data collection, metabolite quantification, and reporting. Additionally, clearer documentation of treatment regimens, study designs, and sample types would help mitigate inconsistencies and provide a more comprehensive understanding of the Trp–Kyn pathway in the context of HIV. Seventh, the majority of participants in each included study were males, which limits the generalization of the findings amongst females or other gender groups. Across a range of demographics, this gender imbalance may lead to an inadequate understanding of the Trp–Kyn pathway. To mitigate this limitation future studies should aim to achieve greater gender diversity by including more females in their research. This approach would provide a better understanding of the Trp–Kyn pathway across a range of demographics. Eighth, the absence of HIV-1 subtypes information in the included studies. Determining the subtype is essential for placing results in context and leaving it out made it impossible to determine whether subtype variation affected the reported Trp–Kyn metabolite levels in PLWH. Reporting HIV-1 subtype information should be a top priority for future studies. This may help provide additional information as to whether differences in the HIV-1 subtype affect the Trp–Kyn pathway metabolite levels in PLWH, this may also reveal whether significant metabolic alterations are specific to certain subtypes. Lastly, we suggest that future studies should refrain from reporting the Kyn/Trp ratio in isolation as an indicator of IDO activity. To more accurately capture IDO-1–mediated immune activation, this ratio should be interpreted alongside key inflammatory markers such as IFN-γ, IL-6, TNF-α and neopterin.

## Conclusions

We conducted a systematic review of studies investigating the Trp–Kyn pathway in PLWH within the modern ART-era. Despite the inherent heterogeneity and limitations of the included studies, the main findings were as follows: Trp, Kyn, and the Kyn/Trp ratio (indicative of IDO activity) were the most commonly investigated metabolites in this field of research. Evidence consistently demonstrated that Trp levels were lower in PLWH, while IDO activity was consistently higher. Despite the widespread investigation of Kyn, there was no clear consensus on its levels in PLWH. These findings suggest that these metabolites and pathways remain active even in participants who are virally suppressed. This highlights their potential as future targets for the development of therapeutics and diagnostics.

## Supplementary Information

Below is the link to the electronic supplementary material.

Supplementary file1 (DOCX 34 KB)

## Data Availability

No datasets were generated or analyzed during the current study.
