# Supplementary material for: The Tryptophan-Kynurenine pathway in people living with HIV: a systematic review
Source: Infection. 2025 May 31;53(5):1625–44. doi: 10.1007/s15010-025-02557-1 (PMC12460365; doi:10.1007/s15010-025-02557-1)
Supplement: Supplementary file 1 — Supplementary file1 (DOCX 34 KB) [file 15010_2025_2557_MOESM1_ESM.docx]

**PubMed**

**25 September 2024**

**Total: 745**

(HIV [tw] OR HIV [mh] OR "aquired immunodeficiency syndrome" [tw] OR AIDS [mh]) AND (tryptophan [tw] OR tryptophan [mh] OR kynurenine [tw] OR kynurenine [mh] OR quinolinic acid [tw] OR quinolinic acid [mh] OR Kynurenic acid [tw] OR kynurenic acid [mh] OR 3-hydroxykynurenine [tw] OR 3-Hydroxyanthranilic Acid [mh] OR picolinic acid [mh] OR picolinic acid [tw] OR anthranilic acid [mh] OR NAD [mh] or Xanthurenic acid [mh])

**Scopus**

**25 September 2024**

**Total: 698**

(HIV OR AIDS) AND ( Tryptophan-Kynurenine AND Pathway)

**Web of Science**

**25 September 2024**

**Total: 1724**

TS=(HIV OR Acquired Immunodeficiency Syndrome OR "Acquired Immunodeficiency Syndrome" OR AIDS) AND TS=(tryptophan OR kynurenine OR quinolinic acid OR Kynurenic acid OR quinaldic acid OR anthranilic acid OR 3-hydroxykynurenine OR xanthurenic acid OR 8-hydroxyquinaldic acid OR 3-hydroxyanthranilic acid OR ɑ-amino-β-carboxymuconate-ε-semialdehyde OR ɑ-aminomuconate- ε-semialdehyde OR picolinic acid OR glutaryl CoA)

**Total: 3167**

**Supplement Table 1: Quality assessment**

| **Quality assessment by TS** | | | | | |
| --- | --- | --- | --- | --- | --- |
| **References** | **Q1** | **Q2** | **Q3** | **Rating** | |
| (Akusjärvi, Krishnan et al. 2023) | 2 | 1 | 2 | 5 | High |
| (Babu, Sperk et al. 2019) | 2 | 1 | 2 | 5 | High |
| (Baer, Colombo et al. 2021) | 2 | 1 | 2 | 5 | High |
| (Chen, Shao et al. 2014) | 1 | 1 | 2 | 4 | Intermediate |
| (Chen, Xun et al. 2019) | 2 | 1 | 1 | 5 | High |
| (Frias, Pagano et al. 2024) | 2 | 2 | 2 | 6 | High |
| (Fuchs, Möller et al. 1991) | 1 | 1 | 1 | 3 | Intermediate |
| (Jenabian, Patel et al. 2013) | 1 | 2 | 2 | 5 | High |
| (Li, Wu et al. 2018) | 2 | 1 | 1 | 4 | Intermediate |
| (Sitole, Tugizimana et al. 2019) | 2 | 2 | 1 | 5 | High |
| (Somsouk, Estes et al. 2015) | 1 | 1 | 2 | 4 | Intermediate |
| (Yang, Cai et al. 2023) | 1 | 1 | 2 | 4 | Intermediate |
| (Yuan, Gan et al. 2023) | 1 | 2 | 1 | 4 | Intermediate |
| **Quality assessment done by LKA** | | | | |  |
| **Reference** | **Q1** | **Q2** | **Q3** | **Rating** |  |
| (Svensson Akusjärvi, Krishnan et al. 2023) | 2 | 2 | 2 | 6 | High |
| (Babu, Sperk et al. 2019) | 2 | 1 | 1 | 4 | Intermediate |
| (Baer, Colombo et al. 2021) | 2 | 1 | 2 | 5 | High |
| (Chen, Shao et al. 2014) | 2 | 1 | 1 | 4 | Intermediate |
| (Chen, Xun et al. 2019) | 2 | 1 | 2 | 5 | High |
| (Frias, Pagano et al. 2024) | 2 | 2 | 1 | 5 | High |
| (Fuchs, Möller et al. 1991) | 1 | 2 | 1 | 4 | Intermediate |
| (Jenabian, Patel et al. 2013) | 2 | 2 | 1 | 5 | High |
| (Li, Wu et al. 2018) | 2 | 1 | 1 | 4 | Intermediate |
| (Sitole, Tugizimana et al. 2019) | 2 | 2 | 1 | 5 | High |
| (Somsouk, Estes et al. 2015) | 2 | 1 | 2 | 5 | High |
| (Yang, Cai et al. 2023) | 2 | 1 | 1 | 4 | Intermediate |
| (Yuan, Gan et al. 2023) | 2 | 2 | 1 | 5 | High |

**Specifically, we assessed the studies with the following questions:**

**1. Were the criteria for sample inclusion clearly defined, and were the study subjects and setting described?**

**2. Was the method for investigating clinical outcomes objective, valid, and reliable?**

**3. Did the study consider confounding factors and employ appropriate statistical analysis?**

**Each question was rated as 0 = no, 1 = partly, or 2 = yes. Studies that answered all the questions with a total rating of ≥5 were classified as high quality. Those with a rating between 3 and 4 were considered intermediate quality, and those with a rating of ≤2 were classified as low quality (see Supplementary Table 1).**
